# The Effect of a One-Day Workshop on the Quality of Framing Multiple Choice Questions in Physiology in a Medical College in India

**DOI:** 10.7759/cureus.44049

**Published:** 2023-08-24

**Authors:** Anup Kumar D Dhanvijay, Nitin Dhokane, Santosh Balgote, Anita Kumari, Ayesha Juhi, Himel Mondal, Pratima Gupta

**Affiliations:** 1 Physiology, All India Institute of Medical Sciences, Deoghar, IND; 2 Physiology, Government Medical College, Sindhudurg, IND; 3 Physiology, Government Medical College, Gondia, IND; 4 Microbiology, All India Institute of Medical Sciences, Deoghar, IND

**Keywords:** all india institute of medical sciences, aiims deoghar, kuder-richardson formula 20, distractor effectiveness, discrimination index, difficulty index, item analysis, mcq, workshop, faculty development program

## Abstract

Background

Multiple choice questions (MCQs) are commonly used in medical exams for more objectivity in assessment. However, the quality of the questions should be optimum for a proper assessment of the students. A faculty development program (FDP) may improve the quality of MCQs. The effect of a one-day workshop on framing MCQ as a part of a FDP has not been explored in our institution.

Aim

This study aimed to evaluate the quality of MCQ in the subject of physiology before and after a one-day workshop on framing MCQ as a part of a FDP.

Methods

This was a retrospective study conducted in the Department of Physiology, All India Institute of Medical Sciences, Deoghar, Jharkhand, India. A one-day workshop on framing MCQ as a part of a FDP was conducted in March 2022. We took 100 MCQs and responses from the students from examinations conducted before the workshop and 100 MCQs and responses from the students after the workshop. In pre-workshop and post-workshop, the same five faculties framed the questions. Post-validation item analysis including difficulty index (DIFI), discrimination index (DI), distractor effectiveness (DE), and Kuder-Richardson Formula 20 (KR-20) for internal consistency was calculated.

Results

Pre-workshop and post-workshop quality of the MCQ remain equal in terms of DIFI (chi-square {3} = 2.42, P = 0.29), DI (chi-square {3} = 2.44, P = 0.49), and DE (chi-square {3} = 4.97, P = 0.17). The KR-20 in pre-workshop and post-workshop was 0.65 and 0.87, respectively. Both had acceptable internal consistency.

Conclusion

The one-day workshop on framing MCQs as a part of a FDP did not have a significant impact on the quality of the MCQs as measured by the three indices of item quality but did improve the internal consistency of the MCQs. Further educational programs and research are required to find out what measures can improve the quality of MCQs.

## Introduction

Assessment is an integral part of the teaching-learning process. In higher education, multiple choice questions (MCQs) are a common tool used to assess students' knowledge and understanding of the subject matter [1]. However, the quality of MCQs has been a matter of concern, as poorly framed questions can lead to erroneous assessment outcomes. Therefore, it is crucial to ensure that the questions are framed accurately, aligning with the learning objectives and assessing higher-order thinking skills [2]. Well-crafted MCQs should not only accurately evaluate the depth of a student's knowledge but also challenge their critical thinking and clinical reasoning abilities. To ensure high quality, MCQs should be based on well-defined learning objectives, relevant to the curriculum, and reflective of real-world medical scenarios [3]. The options provided should be plausible and carefully constructed to avoid ambiguity or bias. Moreover, incorporating distractors that reflect common misconceptions or diagnostic pitfalls can reveal a student's true grasp of the material. Thorough review and validation by subject matter experts are crucial to eliminate errors and ensure that the questions align with the intended learning outcomes [4].

Physiology is a fundamental subject in health sciences taught to first-year medical students, and MCQs are widely used for both formative and summative assessment [5]. However, many faculty members lack formal training in test item writing, leading to poorly constructed questions that may not measure the desired learning outcomes accurately [6]. Therefore, there is a need to improve the quality of MCQs in physiology.

One approach to address this issue is to provide training to faculty members on how to write effective MCQs through a faculty development program (FDP). Studies done in Saudi Arabia and Pakistan showed that one-day FDP can improve the quality of MCQs formed by faculties [7,8]. In contrast, a study from India concluded that a one-day session does not improve the quality of MCQs formed by medical faculty [6]. As the results of different studies are contradicting and the impact of FDP on MCQs has not been explored in the subject of physiology; hence, we conducted this study in our institution.

With this background, the current study aimed to investigate the effect of a one-day workshop on the quality of framing MCQs in physiology. We hypothesized that the workshop would improve the quality of MCQs written by faculty members in physiology. This study's findings may have implications for improving the quality of assessment in physiology and other health sciences subjects.

## Materials and methods

Type and settings

A retrospective study was conducted in the Department of Physiology, All India Institute of Medical Sciences, Deoghar, Jharkhand, India. We obtained the data from the department registries and copies submitted by the students. All the answer sheets were anonymized, and no students' identities were divulged to any author. Fully anonymized data were used for analysis. The study was approved by the Institutional Research Committee (reference number: 2023-103-IND-03).

MCQ

MCQs that were used for the assessment of students in internal semester examinations of physiology in the institution were type A MCQs (i.e., having the single best answer among four options) [9]. During the examinations, the students were instructed clearly on the question paper (written on the top of the question paper) that they needed to mark only the single best answer. In the final analysis, the MCQs that were framed by faculty who participated in the FDP workshop were included.

Workshop

**Figure 1:**
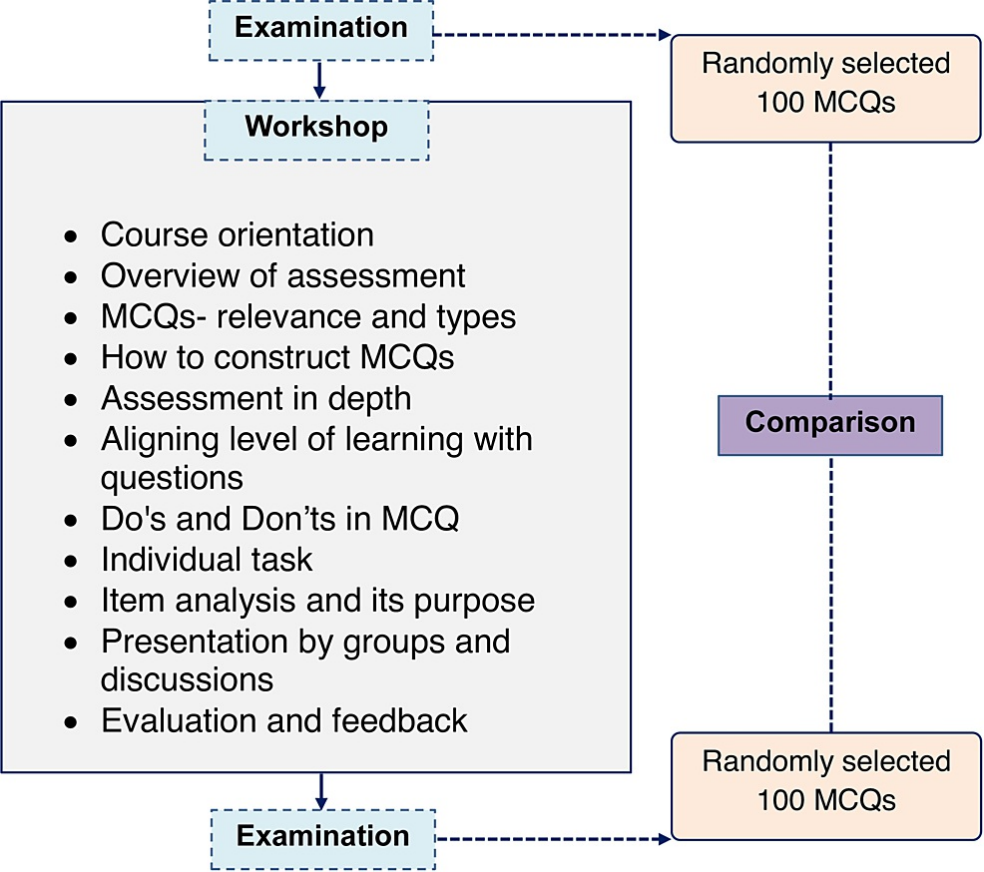
Brief study procedure and workshop plan MCQ: multiple choice question The examinations and workshops were conducted before starting the study. The study only collected data from departmental registries and stored the answer papers of the students.

A full-day workshop was conducted in March 2022 for faculty members of All India Institute of Medical Sciences, Deoghar, Jharkhand, India. The workshop was designed to train faculty members to construct high-quality type A for medical subjects. Five MCQs from each participant's area of expertise were requested so that they may be debated throughout the workshop. Along with the modification of the MCQs structure, theoretical underpinnings were explored in the workshop. All of the faculty members' previously created MCQs for the pre-workshop were altered for improvement. A brief of the workshop and study process is described in Figure 1.

Data and quality indices

**Figure 2:**
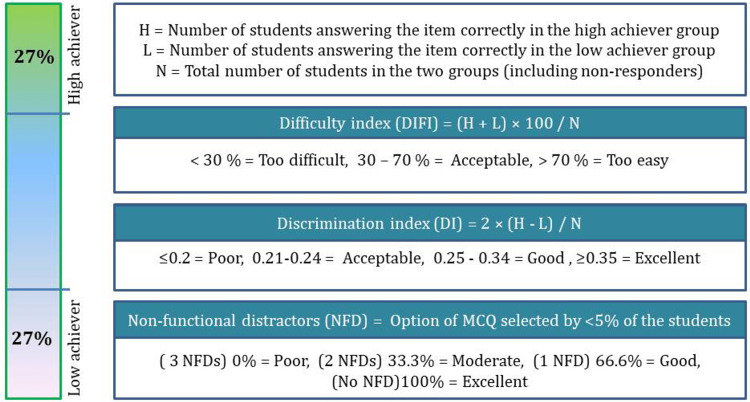
Calculation of DIFI, DI, and non-functional distractors This figure was created by the corresponding author after reviewing relevant literature [12,13].

From the departmental registry, we collected (randomly selected) 100 MCQs set by teachers before and after the workshop. As there is no conclusive guideline for a minimum sample size estimation, we referred to two recently published articles as reference studies where a total of 40 and 90 MCQs were taken for analysis respectively [10,11]. Hence, we decided to include 100 MCQs. All the teachers had >6 years of teaching physiology and formulating questions for undergraduate medical students. Difficulty index (DIFI), discrimination index (DI), distractor effectiveness (DE), and Kuder-Richardson Formula 20 (KR-20) of the MCQs were calculated for pre-workshop and post-workshop MCQs [12,13]. The indices and the range we used to categorize the responses are shown in Figure 2.

Statistical analysis

Microsoft Excel 2010 (Microsoft, Washington USA) was used to generate the database. A chi-square test will be used to compare observed categorical results with expected results. GraphPad Prism 7 (Dotmatics, Boston, Massachusetts, USA) and Microsoft Excel tools were used for the statistical analysis. A p-value of <0.05 will be considered significant.

## Results

We analyzed a total of 100 MCQs before and 100 MCQs after the workshop, and the questions were set by five faculty members who underwent the training in the workshop. The DIFI before and after the workshop are shown in Table 1. There was no significant difference in DIFI before and after the workshop (p = 0.29).

**Table 1:** DIFI of MCQs before and after the workshop DIFI: difficulty index Chi-square (2) = 2.42

DIFI (%)	Pre-workshop	Post-workshop	p-value
<30	33	26	0.29
30-70	47	58	
>70	20	16	

A similar result was also found for DI. There is no significant difference (p = 0.49) between the DI values before and after the workshop (Table 2). This means that the change in DI values observed in the study could be due to chance or random variation rather than the effect of the workshop.

**Table 2:** DI of MCQs before and after the workshop DI: discrimination index Chi-square (3) = 2.44

DI	Pre-workshop	Post-workshop	p-value
≤0.2	22	15	0.49
0.21-0.24	31	28	
0.25-0.34	28	34	
≥0.35	19	23	

A perfect MCQ should have no NFD. Before the workshop, there were eight such MCQs and after the workshop, it increased to 15. However, overall, when the MCQs were categorized into four groups, there was no statistically significant increase (p = 0.17) in the quality according to the DE (Table 3).

**Table 3:** DE of MCQs before and after the workshop NFD: non-functional distractor Chi-square (3) = 4.97

Number of NFD	Pre-workshop	Post-workshop	p-value
Three NFD	35	25	0.17
Two NFD	34	30	
One NFD	23	30	
Zero NFD	8	15	

The KR-20 in pre-workshop and post-workshop was 0.65 and 0.87, respectively. Both had acceptable internal consistency with an increase in the post-workshop phase.

## Discussion

The results of the above study showed that there was no significant difference in DIFI, DI, and DE values before and after the one-day workshop on framing MCQs. This suggests that the workshop did not have a significant impact on the difficulty of MCQs. This finding is consistent with a previous study that also reported limited or no impact of a FDP on the quality of MCQs [6]. However, it should be noted that the overall DIFI values in the present study were within an acceptable range, indicating that the quality of the MCQs was reasonable both before and after the workshop. This is an important finding, as it suggests that the MCQs developed by the faculty members who underwent the training were maintaining quality.

Our study finding contradicts the finding by Alamoudi et al. and Abdulghani et al. who found that FDP improves the quality of MCQ [13,14]. The finding of our study may be attributed to the fact that the workshop was only one day in duration. A longer intervention may be needed to bring about significant improvements in MCQ quality. However, it was beyond the scope of this study. Other factors such as item writing guidelines, item review processes, faculty’s attitude toward the workshop, and adoption willingness may also affect the success of the program.

Beyond academia, the quality of MCQs in medical education holds significant implications. Rigorously designed MCQs that prioritize critical thinking and clinical reasoning skills not only serve as assessments but also shape the preparation of future medical professionals for real-world healthcare challenges [15,16]. Moreover, crafting high-quality MCQs fosters continuous faculty development and complements a broader array of assessment tools, aligning with the trend toward competency-based medical education [17].

Our study highlights the complexity of developing high-quality MCQs and the need for continued education and research on effective MCQ development. While the one-day workshop evaluated in the study showed no significant impact on MCQ quality, other researchers found a single-day workshop to be effective [18,19]. Hence, further research is needed to test other forms of workshops in this institute in the future. Additionally, continued research can help identify best practices for developing high-quality MCQs, such as strategies for constructing effective distractors and improving the discrimination power of questions [20].

Limitation

There are several limitations to this study that should be considered when interpreting the results. The study was conducted at a single institution, which may limit the generalizability of the findings to other settings. The sample size of MCQs was relatively small, which could limit the power to detect significant differences between pre-workshop and post-workshop measures. A total of five faculties formulated the MCQ before and after the workshop. We did not investigate the long-term effects of the workshop on item quality. In addition, the MCQs were set by five faculties of a single department.

## Conclusions

There was no significant difference in DIFI and DI values before and after the workshop. This suggests that the workshop did not have a significant impact on the difficulty and discrimination indices of MCQs. Similarly, the overall quality of NFDs did not show a statistically significant increase after the workshop. However, the internal consistency of the MCQs did improve after the workshop. While the one-day workshop did not have a significant impact on the quality of MCQs, it did improve the internal consistency of the MCQs. However, the findings of the study suggest that further educational programs and research are needed to identify measures that can improve the quality of MCQs.
